# Acupuncture for dry eye: a randomised controlled trial protocol

**DOI:** 10.1186/1745-6215-10-112

**Published:** 2009-12-03

**Authors:** Tae-Hun Kim, Jong-In Kim, Mi-Suk Shin, Myeong Soo Lee, Jun-Yong Choi, So-Yong Jung, Ae-Ran Kim, Jae-Uk Seol, Sun-Mi Choi

**Affiliations:** 1 Clinical Research Centre, Division of Standard Research, Korea Institute of Oriental Medicine, Daejeon, Republic of Korea

## Abstract

**Background:**

Dry eye is usually managed by conventional medical interventions such as artificial tears, anti-inflammatory drugs and surgical treatment. However, since dry eye is one of the most frequent ophthalmologic disorders, safer and more effective methods for its treatment are necessary, especially for vulnerable patients. Acupuncture has been widely used to treat patients with dry eye. Our aim is to evaluate the effectiveness and safety of acupuncture for this condition.

**Methods/Design:**

A randomised, patient-assessor blinded, sham (non-acupuncture point, shallow acupuncture) controlled study was established. Participants allocated to verum acupuncture and sham acupuncture groups will be treated three times weekly for three weeks for a total of nine sessions per participant. Seventeen points (GV23; bilateral BL2, GB4, TE23, Ex1 (Taiyang), ST1 and GB20; and left SP3, LU9, LU10 and HT8 for men, right for women) have been selected for the verum acupuncture; for the sham acupuncture, points have been selected that do not coincide with a classical acupuncture point and that are located close to the verum points, except in the case of the rim of the eye. Ocular surface disease index, tear film breakup time, the Schirmer I test, medication quantification scale and general assessment of improvement will be used as outcome variables for evaluating the effectiveness of acupuncture. Safety will also be assessed at every visit. Primary and secondary outcomes will be assessed four weeks after screening. All statistical analyses will be performed using analysis of covariance.

**Discussion:**

The results of this trial will be used as a basis for clarifying the efficacy of acupuncture for dry eye.

**Trial registration:**

ClinicalTrials.gov NCT00969280.

## Background

Dry eye is one of the most frequently occurring opthalmological health problems worldwide. The prevalence of dry eye is estimated to be in the range of 5% to 35% and its incidence has recently been increasing [1]. According to a recent survey, over 20% of outpatients in ophthalmologic clinics in Korea were diagnosed with dry eye [2]. The burden of dry eye involves not only problems with common activities such as reading, carrying out professional work, using the computer, watching television, and driving [3], but also widespread limitations in the activities of daily life, bodily pain, discomfort and lower energy and vitality [4].

Currently, the use of artificial tears and lifestyle modifications are the most common choices in management of patients with mild dry eye [5]. In moderate and severe cases, anti-inflammatory medications such as corticosteroids, cyclosporine and tetracycline are recommended [6]. However, preservatives in artificial tears may exacerbate ocular surface inflammation and the safety of anti-inflammatory treatment is not well established [6,7]. In this context, various treatments in complementary and alternative medicine (CAM) are administered for dry eye in clinical practice [8]. Acupuncture, one of the most popular CAM interventions, showed some favourable effects over artificial tears for dry eye when administered alone [9,10] or concomitantly [11,12] with artificial tears in several randomised controlled trials (RCTs). The evidence obtained from these trials is quite limited because all of these RCTs were conducted under high risk of bias including uncertainty of blinding, inadequate randomness of allocation of patients, failure to consider sample size and inappropriate control groups. Therefore, well-designed RCTs are needed to establish the efficacy of acupuncture for dry eye.

### Study aims

This study aims to evaluate the effectiveness and safety of acupuncture treatment for dry eye. The null hypothesis of this study is "the change in dry eye symptoms after verum acupuncture treatment is equal to the change in dry eye symptoms after sham acupuncture treatment."

## Methods/Design

**Figure 1:**
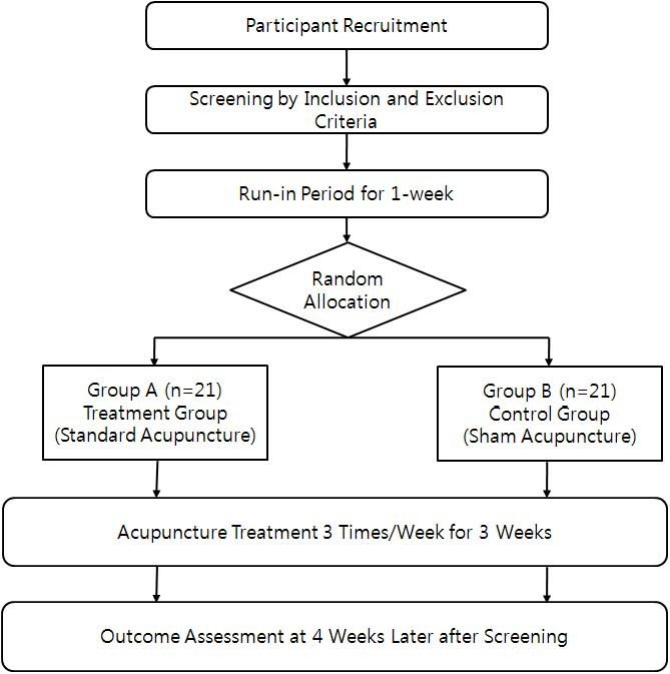
**Study Flow Chart**.

This study is a randomised, patient-assessor blinded, sham acupuncture controlled trial. Enrolled participants will be randomly allocated to either a verum acupuncture or a sham acupuncture group and will receive nine sessions of treatment over a three-week period. Participants will be examined for signs and symptoms of dry eye before and after treatment (Figure 1).

### Recruitment

We will recruit participants by advertisements in local newspapers and on websites of local universities and companies in Daejeon province, Korea. Respondents will be contacted by clinical trial coordinators (CRC) to determine eligibility in a telephone pre-screening. If the applicant is eligible according to the study criteria, he or she will be invited to the clinical research centre of KIOM (Korea Institute of Oriental Medicine) and examined for eligibility by physicians and ophthalmologists.

### Inclusion and exclusion criteria

Eligible participants of both sexes who have moderate to severe levels of dry eye will be enrolled according to the inclusion and exclusion criteria. In addition to abnormal ophthalmological test results (Tear Film Break-Up Time and Schirmer I test), they must have symptoms of dry eye such as ocular itching, ocular foreign body sensation, ocular pain, ocular dryness, or vision problems, which may include blurred vision or a sensation of photophobia. Special medical conditions and conditions that can increase adverse event risk or might be expected to mislead interpretation of the outcome after acupuncture treatment comprise the exclusion criteria (Appendix 1).

### Sample size and randomisation

We wished to estimate the sample size that would suffice to detect an 18 point difference in the Ocular Surface Disease Index (OSDI) between the verum and control groups. In our previous uncontrolled acupuncture study, the standard deviation of OSDI was estimated to be 19.342 [13]. If we apply a two-sided 5% significance level, 90% power and 10% dropout rate, the calculated required sample size is approximately 21 participants in each group, according to the following equation:

An expert on statistics from KIOM who has no direct contact with the study participants will conduct randomisation of participants. He/she will generate random numbers with block randomisation using the SAS statistical package (Version 9.1.3; SAS institute Inc.). The block size will be concealed from other researchers and the randomisation table will not be available for assessment by anyone else involved in the study.

Opaque sealed envelopes containing serial numbers will be delivered to the clinical trial centre. Before the random assignment, all participants will be informed that they will be allocated to one of two groups. Random allocation will be conducted at visit 2. Random numbers with corresponding participants will be determined in the order of the time of second visit and will be opened by acupuncture practitioners. The allocation result will not be announced to the participants until the last visit of the last randomised participant.

The success of blinding will be assessed at each participant's last visit. Researchers who did not participate in the acupuncture treatment and who are blinded to the allocation results will perform the outcome assessment.

### Acupuncture treatment protocol

Acupuncture of both groups will be carried out by licensed oriental medicine physicians who have been trained for four years and have at least five years of clinical experience in acupuncture treatment. Acupuncture practitioners will be required to attend a one-day training course on this study.

### Verum acupuncture group

Standardised acupuncture treatment will be applied to patients in the verum acupuncture group. Acupuncture points were selected according to the opinions of professional acupuncturists in KIOM based on a publication about acupuncture treatment for dry eye [14]. Seventeen acupuncture points represented by bilateral BL2, GB4, TE23, Ex 1 (Taiyang), ST1, and GB20 and unilateral SP3, LU9, LU10, HT8 and GV23 were chosen. For SP3, LU9, LU10, and HT8, males will be treated on the left side and females on the right side according to traditional Korean medicine theory. Disposable, 20 mm*30 mm-size acupuncture needles (Dongbang Co., Korea) will be used. With the exception of Ex1 (Taiyang), each point will be selected and treated in accordance with the WHO Standard Acupuncture Point Locations in the Western Pacific Regional guideline [15]. After insertion, needles will be twisted several times until the participants feel 'de qi' sensation and left for 20 minutes. Each participant will undergo a total of nine sessions (three times per week for three weeks).

### Sham acupuncture group (non-acupuncture point shallow penetration group)

A sham acupuncture point corresponding to each verum acupuncture point was selected. The principle of sham point selection is that the sham point should not be a classical acupuncture point and should, except for points on the rim of the eye, be located close to the verum point. The selected sham points are as follows: both points 2 cm lateral to ST4, 2 cm below ST7, at the parietal eminence of head, 2 cm internal to ST9, 10 cm internal to BL57, 1.5 cm internal to ST36, one point at the left middle point of the bicepsbrachii muscle belly, points at 2 cm, 4 cm, and 6 cm from the Lt. wrist fold, and one point located between the Lt. 3^rd ^and 4^th ^metatarsophalangeal joints. After shallow penetration of the skin at each site with no manipulation for 'de qi,' the needle is left for 20 minutes. Every participant in the sham acupuncture group will be treated three times per week for three weeks.

### Permitted and prohibited concomitant treatments

Participants will be permitted to use artificial tears depending on symptom severity; the frequency and dosage of use must be recorded. However, the use of artificial tears during the two hours prior to acupuncture treatment will be prohibited. Drugs or tear supplements such as cyclosporine, corticosteroids, biological tear substitutes and oestrogen will be prohibited. The wearing of contact lenses will be prohibited during the trial period.

### Primary outcome measurement

OSDI will be used as the primary outcome variable. This questionnaire is a 12-item instrument for assessment of ocular irritation and impact on visual function in dry eye sufferers. It can provide quantifiable results of dry eye symptom frequency and vision-related functioning [16]. Each question will be answered on a scale of 0 to 4, where 0 means none of the time; 1, some of the time; 2, half of the time; 3, most of the time; and 4, all of the time. Each patient's OSDI score will be calculated on the basis of the following formula: OSDI = [(sum of scores for all questions answered)*100]/[(total number of questions answered)*4]. We will use the Korean version of the OSDI questionnaire [17].

### Secondary outcome measurement

The visual analogue scale (VAS) for self-assessment of ocular discomfort will be used. Self-reports on ocular symptoms concomitant with dry eye will be obtained for such conditions as ocular itching, foreign body sensation, burning, pain and dryness as well as for blurred vision, sensation of photophobia, ocular redness and sensation of tearing. Each participant will record a score indicating his or her total level of discomfort on a 100 mm horizontal line.

Tear Film Break-Up Time (TFBUT) and the Schirmer I test with anaesthesia will be used as additional secondary outcome variables. TFBUT is a method for observing tear film stability. Sodium fluorescein (2.5%) will be instilled in both eyes and the tear break-up time (the interval between the last complete blink and the first appearance of a dry spot or disruption in the tear film) will be measured [18]. The Schirmer I test with anaesthesia is a method for checking basic tear secretion. Schirmer test paper (Color Bar™ Eagle Vision, USA) will be inserted on the lateral third of the lower eyelid with the participant's eye closed for five minutes. Each test will be performed by a single ophthalmologist.

Medication Quantification Scale (MQS) and General Assessment of Improvement will also be used as secondary outcome variables. The total frequency and dosage of artificial tears will be checked. At the final visit of each participant, both participant and practitioner will assess improvements in ocular symptoms. 'Excellent' indicates the presence of no symptoms related to dry eye and much improvement in daily life; 'Good' means that a few symptoms related to dry eye are present but that the condition is better than before treatment; 'Fair' means that several symptoms related to dry eye are present but that the condition is a little improved; 'Poor' means that there is no change compared with before treatment; 'Aggravation' means that symptoms are worse than they were before treatment (Table 1).

**Table 1:** Schedule for treatment and outcome measurement

Period	screening										F/U
Visit	1	2	3	4	5	6	7	8	9	10	11
Week		1st			2nd			3rd			Visit10+ 1 week+ 3 days
Informed consent	ν										
Demographic characteristics	ν										
Medical History	ν										
Physical examination	ν										
Random Allocation		ν									
Acupuncture Treatment		ν	ν	ν	ν	ν	ν	ν	ν	ν	
Symptom score	ν	ν	ν	ν	ν	ν	ν	ν	ν	ν	ν
OSDI		ν		ν			ν			ν	ν
Schirmer I test	ν									ν	
TFBUT	ν									ν	
MQS		ν	ν	ν	ν	ν	ν	ν	ν	ν	ν
General assessment (patient/doctor)										ν	
Blinding Assessment		ν									ν
Safety Assessment		ν	ν	ν	ν	ν	ν	ν	ν	ν	ν

OSDI: Ocular Surface Disease Index; TFBUT: Tear Film Break-up Time; MQS: Medication Quantification Scale

### Statistical analysis

We will conduct analysis on an intention-to-treat basis (significance level p < 0.05) using the SAS statistical package program (ver. 9.1.3). Baseline characteristics will be shown as mean ± standard deviation (SD) for continuous data including age, previous duration of dry eye, OSDI, VAS for self-assessment of ocular discomfort, TFBUT and Schirmer's I test value. As for participants' gender, n (%) of male and female in each group will be shown as baseline characteristics. We will conduct between-group comparision in baseline using two-sample t-test or Wilcoxon rank sum test for continuous data and using Chi-square test or Fisher's exact test for gender composition considering p < 0.05 as statistically significant.

For primary and secondary outcome measures, the mean differences from baseline values to the end of treatment will be compared using two-sample t-test or Wilcoxon rank sum test. If any imbalances in baseline characteristics between groups are encountered, we will conduct ANCOVA (analysis of covariance) using these imbalanced variables as covariates and allocated group as fixed factor.

### Adverse events

We define adverse events as unfavourable or unintended signs, symptoms or disease occurring after treatment that are not necessarily related to the acupuncture intervention. Acupuncture-related adverse events are defined as local symptoms including bruising and pain for over two weeks at acupuncture points, peripheral neuritis and swelling, redness and itching for over three days, systemic symptoms including dizziness and palpitation, and psychiatric problems including headache for over three days, anxiety or fear for over 60 hours, and hypersensitivity for over three days. In case of serious medical problems, other symptoms could be included in adverse events. In every visit, adverse events will be reported by participants and examined by the practitioner.

### Ethics

Written consent will be obtained from each participant. This study protocol was approved by the institutional review board of Daejeon University hospital.

## Abbreviations

ANCOVA: analysis of covariance; CAM: complementary and alternative medicine; CRC: clinical trial coordinators; KIOM: Korea Institute of Oriental Medicine; MQS: Medication Quantification Scale; OSDI: Ocular surface disease index; RCTs: randomised controlled trials; SD: standard deviation; TFBUT: Tear Film Break-Up Time; VAS; Visual analogue scale.

## Competing interests

The authors declare that they have no competing interests.

## Authors' contributions

SMC obtained funding for the research project. JIK, MSS, THK drafted the protocol and THK wrote the final manuscript. MSL, JYC, SYJ, ARK and JUS contributed to the research design and made critical revisions. SMC is the representative of KIOM and participated in the trial design as study director. All authors read and approved the final manuscript.

## Appendix 1. Inclusion and Exclusion Criteria

### Inclusion Criteria

Patients who have had dry eye syndrome in a single eye or in both eyes (ICD-10: H04.1).

He or she must have both of the conditions below:

• Patients who have been having symptoms such as ocular itching, ocular foreign body sensation, ocular burning, ocular pain, ocular dryness, blurred vision, sensation of photophobia, ocular redness or sensation of tearing

• Patients whose Tear Film Break-Up Time (TFBUT) is below 10 seconds and measured tear amount is below 10 mm/5 sec by the Schirmer I test

### Exclusion Criteria

• Those who have defects of eyelid or eyelashes

• Those who have acute infection of the eyelid, eyeball or eye accessories

• Those who have Stevens-Johnson syndrome or pemphigoids

• Those who have Vitamin A deficiency

• Those who have any eye or accessory defect due to external injuries

• Those who have undergone any surgical operation related to the eye in the last three months

• Those who are using contact lenses

• Those who have any difficulties of eye opening or eye closing due to facial palsy

• Those who have undergone punctual occlusion surgery

• Those who have used any type of anti-inflammatory eye drops in the last two weeks (steroids, cyclosporin or autologous serum eye drops)

• Those who have systemic immune problems

• Those who are pregnant or plan to become pregnant

• Those who are not appropriate for this study
